# Nasogastric Tube Feeding in Older Patients: A Review of Current Practice and Challenges Faced

**DOI:** 10.1155/2021/6650675

**Published:** 2021-01-21

**Authors:** Devkishan Chauhan, Surabhi Varma, Melanie Dani, Michael B. Fertleman, Louis J. Koizia

**Affiliations:** ^1^Imperial College Healthcare NHS Trust, London, UK; ^2^Cutrale Perioperative and Ageing Group, Imperial College London, London, UK

## Abstract

Nasogastric tube feeding is an essential way of delivering enteral nutrition when the oral route is insufficient or unsafe. Malnutrition is recognised as a reversible factor for sarcopenia and frailty. It is therefore crucial that malnutrition is treated in older inpatients who have dysphagia and require enteral nutrition. Despite five National Patient Safety Alerts since 2005, “Never Events” related to nasogastric feeding persist. In addition to placement errors, current practice often leads to delays in feeding, which subsequently result in worse patient outcomes. It is crucial that tube placement is confirmed accurately and in a timely way. Medical advancements in this area have been slow to find a solution which meets this need. In this paper, we provide an updated review on the current use of feeding nasogastric tubes in the older population, the issues associated with confirming correct placement, and innovative solutions for improving safety and outcomes in older patients.

## 1. Introduction

A feeding nasogastric tube is a flexible, fine-bore, radio-opaque tube passed into the stomach via the nose. It is used to deliver nutritional support and medications to patients who are unable to swallow or are unable to meet their nutritional requirements by mouth. Nasogastric feeding is commonly encountered in older patients (defined as over 65 years old) owing to dysphagia and malnutrition [1]. Dysphagia in older people is very often multifactorial [2]. Causes may be neurological, such as stroke, dementia, and delirium, or mechanical. In addition, poor dentition, reduced moisture in the oral cavity and age-related decline in function of masticatory muscles can compound any swallowing difficulty [3].

Over 790,000 feeding tubes are inserted in the National Health Service (NHS) each year [4]. There are significant challenges that can disproportionately impact older people. This article outlines some of these limitations and identifies innovative solutions to deliver more efficient care to improve patient outcomes.

## 2. Malnutrition

Older patients are a heterogeneous group, with a high prevalence of malnutrition, sarcopenia, and frailty. Malnutrition is recognised as a crucial reversible factor for conditions presenting with sarcopenia and frailty [5]. Older people have an increased risk for comorbid conditions that predispose them to develop malnutrition [6]. The prevalence of nutritional deficiency in the elderly is 15% in ambulatory outpatients, 25%–60% in institutionalised patients, and 35%–65% in hospitalized patients [7]. In order to meet the nutritional needs of this population, clinicians require skills for recognising and managing malnutrition [1].

Malnutrition is not an inevitable side effect of ageing, but many changes associated with the process of ageing can promote malnutrition. For example, ageing is frequently associated with decreases in taste, smell, deterioration in dental health, and decreases in appetite and physical activity [8]. The main body composition changes observed with starvation are a loss of fat-free mass (FFM) and fat mass. In an elderly population, the size of these losses may be greater, as they are in addition to age-related losses in these compartments. The muscle content of the body is greatly important because of its interrelationship with physical function, strength, and morbidity [9].

Enteral feeding is indicated for patients with a functional gastrointestinal tract, whereas parenteral feeding is more commonly used for patients with nonfunctional gastrointestinal tract [10]. In general, enteral nutrition is preferred to parenteral nutrition as it is more physiological, simpler, cheaper, and less complicated [11].

### 2.1. The Challenges of Nasogastric Feeding in the Elderly

There are several challenges that are common with nasogastric tube feeding in the older population. These challenges relate to placement, confirmation of tube placement, dislodgement, and acting in patients' best interest when they lack capacity.

Enteral nutrition has been shown to improve the length of hospital stay and wound healing in selected populations [12, 13]. The benefits of early nasogastric feeding in stroke patients are well established [14]. In addition, Stratton et al. [15] identified that enteral nutrition can reduce the risk of developing pressure ulcers by 25% but the study included enteral tube feeding as well as oral nutritional support [15].

Indications for nasogastric feeding can be broadly divided into two categories: (i) dysphagia (difficulties swallowing) and (ii) anorexia in intercurrent illness or chronic disease. Most evidence for enteral nutrition involves the first category of patients [16]. There is a paucity of studies to evaluate the indications and clinical outcomes of enteral tube feeding in older patients outside the context of poststroke dysphagia and advanced dementia. Due to the impact of multimorbidity and advanced illnesses on malnutrition, there is limited evidence that enteral feeding achieves a sustained response in terms of biochemical markers, weight gain, and other clinical outcomes such as functional status, quality of life, in-patient morbidity, and mortality [17, 18].

Feeding nasogastric tubes can be unpleasant and lead to agitation, particularly in individuals with already established delirium [17]. This leads to an increased risk of dislodgement. Feeding nasogastric tubes can also impair swallowing mechanics prolonging oral [19] and pharyngeal transit times [20], although this is not thought to impact overall swallow function or aspiration risk [21, 22].

Enteral tube feeding in patients with dementia confers no survival benefit or improvement in the quality of life, and there may be an increased risk of mortality [17, 23, 24]. The commonest cause of mortality in nasogastric tube-fed patients is aspiration pneumonia [25]. Enteral nutrition should therefore be carefully considered. The risks and benefits of enteral tube feeding require a holistic assessment and discussion with patients or their advocates. More conservative strategies such as swallow rehabilitation programmes should be considered carefully [3, 26].

### 2.2. Never Events and Other Safety Risks

One of the main risks of blind, bedside nasogastric tube insertion is inadvertent misplacement anywhere along the gastrointestinal tract or the respiratory tract. Feeding or administering medications into the lung is a ‘Never Event' due to potentially fatal harm. Between April 2016 and November 2019, there were 93 ‘Never Events' attributed to feed delivery into the respiratory tract via feeding tubes within the NHS [4]. Five National Patient Safety Alerts have been issued since 2005. However, despite this, and mandating training for the interpretation and communication of X-ray placement, Never Events persist.

Tube intubation into the lung and the pleura carries the risk of pulmonary aspiration, pneumothorax, collapse, or tracheal perforation. Risks of insertion into the gastrointestinal tract include displacement, pulmonary aspiration, oesophagitis, oesophageal stricture, gastrointestinal bleeding, and, very rarely, perforation.

### 2.3. Confirming Placement

#### 2.3.1. Current Practice

NHS Improvement (NHSI) issued guidance on safety-critical requirements for confirming feeding nasogastric tube placement in 2016 [4]. Correct placement can be confirmed by testing the gastric aspirate with a pH strip. Feeding can start only when the pH is 5.5 or below. However, unsuccessful withdrawal of aspirate occurs in 15% of patients [27]. In such cases, where it is not possible to aspirate gastric content or confirm with an acidic pH, verification of tube position is obtained with a chest X-ray. Between 2011 and 2016, 95 incidents were reported to the National Patient Safety Agency (NPSA), and alerts have emphasised the importance of safety checks and interpretation of chest X-rays by trained senior medical staff. The most recent National Patient Safety Alert was released by NHSI in July 2016 [28]. This alert was directed at Trust Boards and not front-line staff as its local investigations found problems with organisational processes for implementing actions from previous alerts. These actions included problems with systems ensuring staff checking tube placement had received competency-based training and that documentation formats included all safety-critical checks.

#### 2.3.2. Is pH a Robust First-Line Test?

Gastric pH is usually 1–5, while respiratory and intestinal pH is usually above 7 [29]. Oesophageal pH is variable due to the influence of refluxed gastric contents and ingested salivary contents [29]. A pH cut-off of 5.5 or less is the practical first-line measure for confirmation of feeding tube placement stipulated in the NHSI guidelines [28]. This is guided by a balance between diagnostic accuracy and cost and utility analysis. It only has a sensitivity of 89% with a specificity of 87% [30]. Analysis of some of the data from the National Reporting and Learning System showed that tube misplacement still occurred in 10 out of 75 cases [31]. At lower cut-off values, there is better differentiation of gastric intubation from oesophageal and pulmonary placement [32, 33]. However, lowering the threshold would increase the number of false negatives, requiring more x-rays for verification [32]. The consequences of this include unnecessary radiation exposure to the patient and a delay in the time to start feeding.

It is estimated that, in almost 45% of patients, it is not possible to obtain an aspirate in the first instance [33, 34]. There is also a risk of operator variability in the interpretation of pH strips, and inability if suffering from colour blindness [35]. In addition, falsely raised pH is associated with acid-suppression therapy. Studies have found that one in nine older patients are on longer-term proton pump inhibitors [29, 36, 37]. These factors preclude pH testing as an effective confirmation test in a significant proportion of older people.

#### 2.3.3. Limitations of X-Ray Confirmation

**Figure 1:**
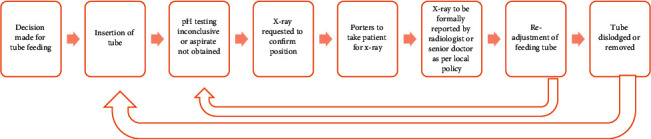
Schematic representing multistep process of feeding tube placement confirmation that leads to delays.

As a result of the limitations described with pH testing, X-ray is commonly required to confirm correct placement. When the appropriate safety checks by a trained clinician are adhered to, X-ray confirmation of placement is a safe and robust mechanism. However, a study looking at feeding tube placements in stroke patients demonstrated that tube insertion occurred within 2.5 hours of request but the additional time required for X-ray confirmation led to a delay in using the tube of 8–9 hours per case [38].  Figure 1 depicts the typical delays that are encountered from a decision being made about enteral feeding, to the start of feeding.

### 2.4. Cost Implications for Feeding Tube Confirmation

The overall financial cost of feeding tube placement confirmation is unknown. Simplistically, pH strips cost as little as £0.07 and the results can be instantly interpreted by a trained nurse, whereas X-rays cost between £50- and £75 and formal reporting by a radiologist are usually required. But the true cost could spiral when clinician, radiographer, and portering time is taken into account, especially when considering the impact of delayed nutrition on the patient.

Few studies have evaluated the true financial cost of feeding tube placement. McFarland [39] set out to “evaluate the effectiveness of pH paper testing of aspirate and chest X-ray for determining nasogastric tube placement in terms of cost and patient outcome” [40]. In this study, the costs of a pH test are estimated to be £43.20 and X-ray £158.64. If this is extrapolated, a conservative estimate based on numbers inserted per year in the UK alone would be more than £30 million.

In addition, the financial cost can be further explored by assessing the impact of complications from misplacement. Aguilar-Nascimento et al. [40] identified misplaced tubes were more likely to expedite a stay in intensive care [40].

### 2.5. Dislodgement

Dislodgement of feeding is common and ranges from 25 to 82% of patients [38, 41]. Given the prevalence of delirium and chronic neurological disorders in older people, dislodgement of feeding tubes is a common occurrence, thus increasing the risks of adverse events and aspiration to the patient.

In individuals where tube feeding is considered essential and in their best interests and dislodgement is feared, the benefits of some forms of restraint should be considered. This may include retaining devices or mittens. One to one (specialing) supervision is less restrictive and should always be attempted in the first instance, as all forms of restraint are examples of deprivation of liberty. The use of physical restraints can hinder the promotion of self-reliance and can impact the individuals' autonomy and dignity [42]. Both the Care Quality Commission (CQC) (United Kingdom Department of Health) and National Institute for Health and Care Excellence (NICE—United Kingdom) state that restrictive interventions should be minimized and used for the shortest amount of time [43, 44].

Retaining systems utilise an anchor device, usually inserted via a stylet and a probe with magnetic ends that loop around the vomer bone or the nasal septum. The ends are tied to the nasogastric tube. Bridles reduce the rate of tube removal, increase caloric feeding, and minimise the use of resources [45–47]. Their use has not become common practice due to the perception of reduced tolerability and risk of skin ulceration, traumatic epistaxis, and nasal rupture [46]. There is no evidence yet to suggest a statistically significant difference in adverse events [47].

No studies have looked at the efficacy of mittens or 1 : 1 observational measures in tube securement, although an observational study reported that patients with mittens were eight times more likely to remove the tube [38]. This may be confounded by the fact that mittens are usually reserved for very agitated, confused patients at the highest risk of tube removal.

### 2.6. Potential Solutions and Innovative Options for Placement Checks

Recent times have seen tremendous advancements in medical technology and innovation. Artificial intelligence, machine learning, and virtual reality have led to a transformation in the way healthcare is delivered [48]. However, despite persistent patient safety concerns and the challenges of obtaining a timely confirmation of tube placement there has been a paucity in the development of placement checks.

The existing practice is archaic and a breakthrough to transform feeding tube placement confirmation is well overdue. We have highlighted the areas of development which are being pioneered and may lead to solutions and transformation of how checks could develop.

#### 2.6.1. Alternatives to Current Standards of pH Testing

The British Gastroenterology Society lent support to the evidence that the pH cut-off should be 5 or below to improve diagnostic accuracy [31, 41]. This however would lead to an increase in false negative results and an increased need for x-rays. This would lead to additional delays without necessarily improving patient care. If this change were to be introduced it would further substantiate our calls for live bedside imaging to confirm placement checks.

Alternatively, other gastrointestinal biomarkers such as bilirubin and digestive enzymes as an adjunct to routine pH testing have been shown to detect misplacement. [30, 49, 50]. The development of commercially viable test-kits requires an understanding of the stability, storage, and buffering of these reagents for calorimetric application. However, it provides scope for practical, point of care testing if further research supports its improved diagnostic value compared to pH alone. The main issue relating to these measures, however, is that they rely on an aspirate, which can be difficult to obtain as mentioned above.

#### 2.6.2. Alternatives to Aspirate Analysis

A possible solution to obtaining an aspirate is direct intragastric detection of pH via the nasogastric tube. A small study has shown successful placement of nasointestinal tubes attached to a pH sensor, guided by a change in pH readings measured by an external pH monitor [51]. This is a promising innovation which may prove to provide an accurate and reliable point of care test. However, the fibre optic sensor would need to be reinserted each time a pH check is required.

With the establishment of catheter-based intraoesophageal monitoring in the evaluation of gastrooesophageal reflux disease over the past decade, its potential for adaptation to facilitate a probe that continually records the pH to an external monitor offers an avenue to explore.

#### 2.6.3. Alternatives to X-Rays

Real-time tracking or visualisation of the feeding tube as it travels from the nasal cavity into the stomach would allow timely identification of any misplacement, immediate confirmation of nasogastric placement, and, thus, earlier initiation of feeding. This would be ideal and prevent potential misplacement. By the time the tube is placed, the X-ray performed and interpreted the damage may have already occurred particularly with regard to a pneumothorax.

Bedside imaging using ultrasound guided insertion and electromagnetic placement devices have been trialled [52]. These approaches require highly technical skills in interpretation. The equipment can be bulky and often inappropriate for bedside evaluation. The Kangaroo feeding tube uses real-time imaging through an intraluminal camera which is less than 3 mm and fits within the tube. This mechanism has proved to be beneficial but would require expert training and prove to be a costly alternative.

A colorimetric capnograph is a device designed to detect carbon dioxide gas; changing levels of gas results in a change in the colour of the device. Meyer et al. [53] found capnography was able to accurately ensure correct gastric tube insertion [53]. The only limitation is that the majority of studies look at capnography in critically unwell intubated patients. More research is required (Table 1).

## 3. Conclusion

Nasogastric tube feeding is a common form of short-term enteral feeding in older people and has clear benefits in selected patients. The confirmation of placement is of critical importance and the existing process requires an innovative solution to transform current practice. This article highlights the inadequacies that exist with the current methods of confirming feeding tube placement, particularly in frail patients, and the delays to feeding and patient harm that occur as a result. Hospital guidelines should emphasise the expeditious safety checks by a senior clinician, and local protocols should focus on minimising delays in starting feeding. There remains an urgent need to direct future research and biotechnological advances into making nasogastric tubes safer, easier to tolerate, and simpler to use.

## 4. Clinical Implications

  Nasogastric tube feeding is a common form of short-term enteral feeding in older people
  Current methods of confirming tube placement, particularly in frail patients, results in delays in feeding, and patient harm can occur as a result.
   Confirmation of tube placement is of critical importance and the existing process requires an innovative solution to transform current practice.
   There remains an urgent need to direct future research and biotechnological advances into making nasogastric tubes safer, easier to tolerate, and simpler to use.

## Figures and Tables

**Table 1:** Summary of the various techniques that have been explored (✔pros, ✖cons).

Novel strategies to enable “real-time” confirmation of nasogastric placement	
Ultrasound	An alternative technique for live imaging is through ultrasound (US). The feeding tube can also be visualised at the gastrooesophageal junction with longitudinal and angled US scans of the epigastrium. Various studies support the use of US [54]. ✔ Reduced delay in administering enteral nutrition ✖ Requiring appropriately trained individuals ✖Availability of US scanners ✖Lack of studies looking at adverse events [55]

Electromagnetic placement devices	CORTRAK 2 enteral access system has received FDA approval. The nasogastric catheter has an electromagnetic coil at the end. As the tube is advanced, a receiver unit placed externally along the xiphoid process captures electromagnetic signalling and converts it into a 3-dimensional image of the tube tip relative to the diaphragm. This is displayed on a monitor. ✔Comparable accuracy in confirming NG positioning compared to X-ray [56,57] ✔Reduced delay in administering enteral nutrition ✔Real-time visualisation shown to reduce misplacement [52] ✖ Real-world data has highlighted the risk of pulmonary complications. The FDA issued an alert regarding 51 medical device reports about pneumothorax related events, including 11 patient deaths between 2012 and 2017 ✖ The majority of adverse events are due to misinterpretation of tracings by the operator highlighting the expertise and skill required for this device ✖ Estimated additional cost of £70-£76 per treatment session. Thus, it has not been approved by NICE, unless there is evidence that it obviates the need for X-rays and pH testing.

Integrated real-time imaging system (IRIS) technology	The kangaroo feeding tube (Covidien Commercial ltd.) has an IRIS in the form of a 3 mm camera attached to the end of the feeding tube, which allows direct visualisation of anatomical landmarks. It has received FDA approval and is currently authorised for research use in the UK. A prospective study on 21 patients in a neurological ITU showed that 90% of patients were successfully intubated, and correct placement was confirmed in all, using abdominal X-ray with contrast [58] ✔Real-time tracking ✔ Scope for the camera to verify placement regularly, prior to enteral feeding, and reduce the need for check X-rays. ✖ It is anticipated that outcomes will be operator-driven, but the training expertise required needs to be evaluated. Further studies are needed to ascertain safety, accuracy, cost-efficiency, and camera visualisation performance and endurance.

Magnetic technology	A small magnet at the distal end of the nasogastric tube can be used to drag its placement infradiaphragmatically and through the transpyloric sphincter, using an external magnet. Successful transpyloric placement has been demonstrated ✖ Does not enable real-time localisation ✖ Hand-held external magnets can be difficult to use in a ward environment with other magnetic sources contraindicated in patients with implantable cardiac devices

Passive magnetic localisation technology using magnetic sensors	The magnetic tip can be traced by the use of stationary electric sensors positioned externally on the body. An experimental study used a cervical device embedded with magnetic sensors to differentiate the trajectory of the nasogastric catheters in the cervical oesophagus from the trachea, ex vivo [59]. Improved sensitivity of magnetic sensors and improved cost-efficiency provide favourable incentive to develop this technology further

## References

[1] Mundi M. S., Patel J., McClave S. A., Hurt R. T. (2018). Current perspective for tube feeding in the elderly: from identifying malnutrition to providing of enteral nutrition. *Clinical Interventions in Aging*.

[2] Smukalla S. M., Dimitrova I., Feintuch J. M., Khan A. (2017). Dysphagia in the elderly. *Current Treatment Options in Gastroenterology*.

[3] Sura L., Madhavan A., Carnaby G., Crary M. A (2012). Dysphagia in the elderly: management and nutritional considerations. *Clinical Interventions in Aging*.

[4] Improvement NHS, Patient Safety Alert, 2016, https://improvement.nhs.uk/documents/177/Patient_Safety_Alert_Stage_2_-_Deterioration_resources_July_2016_v2.pdf

[5] Churchward-Venne T. A., Breen L., Phillips S. M. (2014). Alterations in human muscle protein metabolism with aging: protein and exercise as countermeasures to offset sarcopenia. *Biofactors*.

[6] Brownie S. (2006). Why are elderly individuals at risk of nutritional deficiency?. *International Journal of Nursing Practice*.

[7] Vellas B., Lauque S., Andrieu S. (2001). Nutrition assessment in the elderly. *Current Opinion in Clinical Nutrition and Metabolic Care*.

[8] Hickson M. (2006). Malnutrition and ageing. *Postgraduate Medical Journal*.

[9] Reed R. L., Yochum K., Pearlmutter L., Meredith K. E., Mooradian A. D. (1991). The interrelationship between physical exercise, muscle strength and body adiposity in a healthy elderly population. *Journal of the American Geriatrics Society*.

[10] Wanten G., Calder P. C., Forbes A. (2011). Managing adult patients who need home parenteral nutrition. *BMJ*.

[11] Masaki S., Kawamoto T. (2019). Comparison of long-term outcomes between enteral nutrition via gastrostomy and total parenteral nutrition in older persons with dysphagia: a propensity-matched cohort study. *PLoS One*.

[12] Herbert G., Perry R., Andersen H. K. (2018). Early enteral nutrition within 24 hours of lower gastrointestinal surgery versus later commencement for length of hospital stay and postoperative complications. *Cochrane Database of Systematic Reviews*.

[13] Schroeder D., Gillanders L., Mahr K., Hill G. L. (1991). Effects of immediate postoperative enteral nutrition on body composition, muscle function, and wound healing. *Journal of Parenteral and Enteral Nutrition*.

[14] Dennis M., Lewis S., Cranswick G. (2006). FOOD: a multicentre randomised trial evaluating feeding policies in patients admitted to hospital with a recent stroke. *Health Technol Assess*.

[15] Stratton R. J., Ek A.-C., Engfer M. (2005). Enteral nutritional support in prevention and treatment of pressure ulcers: a systematic review and meta-analysis. *Ageing Research Reviews*.

[16] Ojo O., Brooke J. (2016). The use of enteral nutrition in the management of stroke. *Nutrients*.

[17] Ciocon J. O., Silverstone F. A., Graver L. M. (1988). Tube feedings in elderly patients. *Archives of Internal Medicine*.

[18] Nesemeier R., Dunlap N., McClave S. A. (2017). Evidence-based support for nutrition therapy in head and neck cancer. *Current Surgery Reports*.

[19] Wang T.-G., Wu M.-C., Chang Y.-C., Hsiao T.-Y., Lien I.-N. (2006). The effect of nasogastric tubes on swallowing function in persons with dysphagia following stroke. *Archives of Physical Medicine and Rehabilitation*.

[20] Wang Z.-Y., Chen J.-M., Ni G.-X. (2019). Effect of an indwelling nasogastric tube on swallowing function in elderly post-stroke dysphagia patients with long-term nasal feeding. *BMC Neurol*.

[21] Dziewas R., Warnecke T., Hamacher C. (2008). Do nasogastric tubes worsen dysphagia in patients with acute stroke?. *BMC Neurology*.

[22] Jaafar M. H., Mahadeva S., Morgan K., Tan M. P. (2015). Percutaneous endoscopic gastrostomy versus nasogastric feeding in older individuals with non-stroke dysphagia: a systematic review. *The Journal of Nutrition, Health & Aging*.

[23] Rudberg M. A., Egleston B. L., Grant M. D., Brody J. A. (2000). Effectiveness of feeding tubes in nursing home residents with swallowing disorders. *Journal of Parenteral and Enteral Nutrition*.

[24] Ayman A. R., Khoury T., Cohen J. (2017). PEG insertion in patients with dementia does not improve nutritional status and has worse outcomes as compared with PEG insertion for other indications. *Journal of Clinical Gastroenterology*.

[25] Gomes G. F., Pisani J. C., Macedo E. D., Campos A. C. (2003). The nasogastric feeding tube as a risk factor for aspiration and aspiration pneumonia. *Current Opinion in Clinical Nutrition and Metabolic Care*.

[26] Chou H.-H., Tsou M.-T., Hwang L.-C. (2020). Nasogastric tube feeding versus assisted hand feeding in-home healthcare older adults with severe dementia in Taiwan: a prognosis comparison. *BMC Geriatrics*.

[27] Neumann M. J., Meyer C. T., Dutton J. L., Smith R. (1995). Hold that X-ray. *Journal of Clinical Gastroenterology*.

[28] NHS Improvment (2016). Patient safety alert: nasogastric tube misplacement: continuing risk of death and severe harm. *NHS Improvment*.

[29] Metheny N. A., Titler M. G. (2001). Assessing placement of feeding tubes. *American Journal of Nursing*.

[30] Metheny N. A., Stewart B. J., Smith L., Yan H., Diebold M., Clouse R. E. (1999). pH and concentration of bilirubin in feeding tube aspirates as predictors of tube placement. *Nursing Research*.

[31] Ni M. Z., Huddy J. R., Priest O. H. (2017). Selecting pH cut-offs for the safe verification of nasogastric feeding tube placement: a decision analytical modelling approach. *BMJ Open*.

[32] Fernandez R. S., Chau J. P.-C., Thompson D. R., Griffiths R., Lo H.-S. (2010). Accuracy of biochemical markers for predicting nasogastric tube placement in adults-A systematic review of diagnostic studies. *International Journal of Nursing Studies*.

[33] Taylor S. J., McWilliam H., Allan K., Hocking P. (2015). The efficacy of feeding tubes: confirmation and loss. *British Journal of Nursing*.

[34] Borsci S., Buckle P., Huddy J. (2017). Usability study of pH strips for nasogastric tube placement. *PLoS One*.

[35] Rowat A. M., Graham C., Dennis M. (2018). Study to determine the likely accuracy of pH testing to confirm nasogastric tube placement. *BMJ Open Gastroenterol*.

[36] Wallerstedt S. M., Fastbom J., Linke J., Vitols S. (2017). Long-term use of proton pump inhibitors and prevalence of disease- and drug-related reasons for gastroprotection-a cross-sectional population-based study. *Pharmacoepidemiology and Drug Safety*.

[37] Rotman S. R., Bishop T. F. (2013). Proton pump inhibitor use in the U.S. ambulatory setting, 2002-2009. *PLoS One*.

[38] Brazier S., Taylor S. J., Allan K., Clemente R., Toher D. (2017). Stroke: ineffective tube securement reduces nutrition and drug treatment. *British Journal of Nursing*.

[39] McFarland A. (2017). A cost utility analysis of the clinical algorithm for nasogastric tube placement confirmation in adult hospital patients. *Journal of Advanced Nursing*.

[40] De Aguilar-Nascimento J. E., Kudsk K. A. (2007). Clinical costs of feeding tube placement. *Journal of Parenteral and Enteral Nutrition*.

[41] Stroud M., Duncan H., Nightingale J. (2003). Guidelines for enteral feeding in adult hospital patients. *Gut*.

[42] Wang W. W., Moyle W. (2005). Physical restraint use on people with dementia: a review of the literature. *The Australian Journal of Advanced Nursing: A Quarterly Publication of the Royal Australian Nursing Federation*.

[43] Care Quality Commission (2015). Brief guide: restraint (physical and mechanical). https://www.cqc.org.uk/sites/default/files/20180322_900803_briefguide-restraint_physical_mechanical_v1.pdf.

[44] National Institute for Health and Care Excellence (2018). Dementia: assessment, management and support for people living with dementia and their carers. *Grants Register*.

[45] Taylor S. J., Allan K., Clemente R., Marsh A., Toher D. (2018). Feeding tube securement in critical illness: implications for safety. *British Journal of Nursing*.

[46] Seder C. W., Stockdale W., Hale L., Janczyk R. J. (2010). Nasal bridling decreases feeding tube dislodgment and may increase caloric intake in the surgical intensive care unit: a randomized, controlled trial. *Critical Care Medicine*.

[47] Brugnolli A., Ambrosi E., Canzan F., Saiani L. (2014). Securing of naso-gastric tubes in adult patients: a review. *International Journal of Nursing Studies*.

[48] Martin G., Koizia L., Kooner A. (2020). Use of the HoloLens2 mixed reality headset for protecting health care workers during the COVID-19 pandemic: prospective, observational evaluation. *Journal of Medical Internet Research*.

[49] Metheny N. A., Smith L., Stewart B. J. (2000). Development of a reliable and valid bedside test for bilirubin and its utility for improving prediction of feeding tube location. *Nursing Research*.

[50] Metheny N. A., Stewart B. J., Smith L., Yan H., Diebold M., Clouse R. E. (1997). pH and concentrations of pepsin and trypsin in feeding tube aspirates as predictors of tube placement. *JPEN. Journal of Parenteral and Enteral Nutrition*.

[51] Heiselman D. E., Vidovich R. R., Milkovich G., Black L. D. (1993). Nasointestinal tube placement with a pH sensor feeding tube. *Journal of Parenteral and Enteral Nutrition*.

[52] Smithard D., Barrett N. A., Hargroves D., Elliot S. (2015). Electromagnetic sensor-guided enteral access systems: a literature review. *Dysphagia*.

[53] Meyer P., Henry M., Maury E., Baudel J.-L., Guidet B., Offenstadt G. (2009). Colorimetric capnography to ensure correct nasogastric tube position. *Journal of Critical Care*.

[54] Brun P.-M., Chenaitia H., Lablanche C. (2014). 2-Point ultrasonography to confirm correct position of the gastric tube in prehospital setting. *Military Medicine*.

[55] Tsujimoto H., Tsujimoto Y., Nakata Y., Akazawa M., Kataoka Y. (2017). Ultrasonography for confirmation of gastric tube placement. *Cochrane Database of Systematic Reviews*.

[56] Taylor S., Allan K., McWilliam H. (2014). Confirming nasogastric tube position with electromagnetic tracking versus pH or X-ray and tube radio-opacity. *British Journal of Nursing*.

[57] Powers J., Luebbehusen M., Spitzer T. (2011). Verification of an electromagnetic placement device compared with abdominal radiograph to predict accuracy of feeding tube placement. *Journal of Parenteral and Enteral Nutrition*.

[58] Mizzi A., Cozzi S., Beretta L., Greco M., Braga M. (2017). Real-time image-guided nasogastric feeding tube placement: a case series using Kangaroo with IRIS Technology in an ICU. *Nutrition*.

[59] Sun Z., Foong S., Maréchal L., Tan U.-X., Teo T. H., Shabbir A. (2015). A non-invasive real-time localization system for enhanced efficacy in nasogastric intubation. *Annals of Biomedical Engineering*.

